# High‐dose steroid therapy for acute respiratory distress syndrome lacking common risk factors: predictors of outcome

**DOI:** 10.1002/ams2.321

**Published:** 2017-10-25

**Authors:** Yoshiaki Kinoshita, Hiroshi Ishii, Hisako Kushima, Kentaro Watanabe, Masaki Fujita

**Affiliations:** ^1^ Department of Respiratory Medicine Fukuoka University Hospital Fukuoka Japan; ^2^ General Medical Research Centre Fukuoka University School of Medicine Fukuoka Japan

**Keywords:** Computed tomography, consolidation, diffuse alveolar damage, ground‐glass attenuation, high‐dose methylprednisolone pulse therapy

## Abstract

**Aim:**

Acute respiratory distress syndrome (ARDS) is a life‐threatening lung disease that usually occurs in patients with the underling risk factors that triggers lung inflammation. We sometimes encounter patients with ARDS lacking common risk factors. Recent studies have indicated the effectiveness of corticosteroids for this cohort. However, the characteristics of survivors with ARDS who lack common risk factors, and who received high‐dose methylprednisolone pulse therapy (MPPT), are not known.

**Methods:**

We undertook a retrospective study of patients with ARDS lacking common risk factors, who received i.v. MPPT for 3 days. The patients (*n* = 46) were classified into two groups, survivors (*n* = 23) and non‐survivors (*n* = 23), based on their survival at 60 days after the initiation of MPPT, and their clinical and radiological parameters were evaluated.

**Results:**

The patient characteristics and disease severity of the two groups were comparable. The percentage of consolidation/(ground‐glass attenuation [GGA] + consolidation) on the chest computed tomography scans of survivors was significantly lower than that of non‐survivors (survivors, 5.63% [2.31–13.8] versus non‐survivors, 27.2% [5.97–41.4]; *P *=* *0.01). In the stratified analysis, the percentage of consolidation/(GGA + consolidation) was significantly associated with 60‐day survival.

**Conclusions:**

Our results show that the percentage of consolidation/(GGA + consolidation) on the chest CT scans is an independent prognostic factor for patients with ARDS lacking common risk factors after MPPT.

## Introduction

The acute respiratory distress syndrome (ARDS) is a life‐threatening inflammatory lung disease with a mortality rate of 40–50%.^1^, ^2^ Acute respiratory distress syndrome is defined as acute onset of hypoxemia and bilateral pulmonary infiltrates not attributed to cardiac failure or fluid overload with pulmonary or non‐pulmonary risk factors that trigger lung inflammation.^1^, ^2^ Diffuse alveolar damage (DAD), which is usually resistant to treatment, is the accepted histological hallmark of ARDS.^1^, ^2^

We sometimes encounter patients with ARDS who lack the common risk factors. A retrospective study showed that 7.5% of ARDS had no risk factor identified, and they included four etiological categories: autoimmune (36%), drug‐induced (26%), malignant (14%), and idiopathic (24%).^3^ In addition, this cohort may include several histological types of lung injury other than DAD, such as non‐specific interstitial pneumonia, organizing pneumonia, diffuse alveolar hemorrhage, hypersensitivity pneumonia, and eosinophilic pneumonia.^4^, ^5^, ^6^ The responsiveness of these patients to corticosteroids differs to that of pure ARDS/DAD patients.^3^, ^4^, ^5^, ^6^ Corticosteroid therapy for the treatment of ARDS lacking common risk factors is therefore worthy of consideration.

Intravenous high‐dose methylprednisolone pulse therapy (MPPT) has sometimes been used for the treatment of patients with the acute pulmonary injury of unknown cause. Although the effectiveness of corticosteroids in ARDS patients remains controversial or lacking in evidence,^7^, ^8^, ^9^ previous studies supported the clinical responsiveness of corticosteroids to ARDS lacking common risk factors.^3^, ^4^, ^6^, ^8^ However, we should be prudent in the use corticosteroids for lung injury from the perspective of complications, including systemic serious infection, hyperglycemia, hypertension, thrombosis, and neuromuscular dysfunction.^7^ The aim of the present study is to identify the characteristics of survivors with ARDS lacking common risk factors, who received i.v. MPPT.

## Methods

### Subjects

We retrospectively reviewed the medical records of consecutive patients with respiratory failure who were admitted to our department from November 2008 to November 2015. The inclusion criteria specified patients who: were 18 years of age and older; met the Berlin definition of ARDS^1^ excluding common risk factors; received i.v. MPPT (0.5–1 g/day) for 3 days; underwent chest computed tomography (CT) within 3 calendar days of MPPT; and whose laboratory profiles were analyzed within 24 h of the initiation of MPPT. In this study, ARDS was diagnosed according to the Berlin definition based on the PaO_2_/F_I_O_2_ (P/F) ratio with a positive end‐expiratory pressure or continuous positive airway pressure of ≥5 cmH_2_O.^1^ However, patients who did not receive positive‐pressure mechanical ventilation but who had a P/F ratio of ≤300 were included in the present study. If the patient was not supported by a mechanical ventilator, the F_I_O_2_ level was calculated as follows: oxygen flow (L/min) × 0.03 + 0.21.^10^ The common risk factors for ARDS were defined as aspiration, diffuse pulmonary infection, near‐drowning, toxic inhalation, lung contusion, non‐pulmonary sepsis, severe non‐thoracic trauma, pancreatitis, severe burns, non‐cardiogenic shock, drug overdose, hypertransfusion for emergency resuscitation, and cardiopulmonary bypass.^1^, ^2^

The exclusion criteria were as follows: pre‐existing interstitial pneumonia; chronic respiratory failure requiring home oxygen therapy; malignancy that was likely to result in death within 6 months; or concomitant pneumothorax on admission. The patients were classified into two groups, survivors and non‐survivors, based on their survival at 60 days after the initiation of MPPT. The Fukuoka University Hospital Institutional Review Board (Fukuoka, Japan) approved the study protocol and waived the requirement for informed consent (approval number: 16‐1‐15).

### Intravenous high‐dose MPPT

In our department, MPPT has sometimes been considered as a treatment option for patients with acute pulmonary injury, when they had a P/F ratio of ≤300 but did not have any apparent cause. In particular, infectious pneumonia was ruled out before receiving MPPT to the extent that was possible. Methylprednisolone pulse therapy was followed by a tapered dosage of prednisolone in 41/46 (89.1%) patients. The tapering schedule of prednisolone varied among individuals.

### Clinical characteristics

The following data were abstracted from the medical records: patient characteristics, oxygenation parameters of the P/F ratio, scoring systems of Acute Physiology and Chronic Health Evaluation II (APACHE II) and Sequential Organ Failure Assessment (SOFA), laboratory profiles, complications, concurrent therapies, cause of death, and (if available) histocytological appearance. The P/F ratio and the scoring systems of APACHE II and SOFA were calculated within 24 h of the initiation of MPPT. In the calculation of the scores, the worst values for each parameter in the 24‐h period were used.

### Etiologies of lung injury

The etiologies of ARDS lacking common risk factors were examined before or after the initiation of MPPT. The diagnosis of connective tissue disease‐associated interstitial lung disease (CTD‐ILD) was based on the current criteria for each of the CTDs.^11^, ^12^, ^13^, ^14^, ^15^ Drug‐induced lung injury was diagnosed when patients met the definition of probable drug‐induced lung injury according to the criteria proposed by Dhokarh *et al*.^16^ In addition, if the etiologies of lung injury were not determined at the time of death or during the follow‐up, the etiology was evaluated as “unknown”.

### Imaging data

If chest CT was carried out several times within 3 calendar days of MPPT, we evaluated the chest CT that was undertaken at the nearest time from the initiation of MPPT. Chest CT scans were independently evaluated by two pulmonologists (HI and YK), with 22 and 11 years of experience in chest CT image interpretation, respectively.

The CT variables that were assessed included the extent of ground‐glass attenuation (GGA) and consolidation and the presence of bronchial dilation, honeycombing, interlobular septal thickening, and pleural effusion. Each CT finding was defined according to the Fleischner Society glossary of terms.^17^ Pleural effusion was defined based on the presence of pleural fluid with a thickness of ≥1 cm from the parietal pleura. According to the previous methods regarding the extent of the lung opacity,^18^ each lung was divided into upper, middle, and lower zones. The extent of each abnormality was determined in accordance with the visually estimated percentage (to the nearest 5%) of lung opacity/total lung area in each zone, and the percentages of lung opacity in each zone were averaged. The extent (as a percentage) of each lung opacity was averaged between the two observers. Disagreements regarding the presence of each CT finding were resolved by consensus.

### Statistical analysis

Continuous data are shown as the group median (interquartile range), and categorical data are shown as the number (percentage) in the group. Fisher's exact test was used to compare categorical variables. The Mann–Whitney *U*‐test was used to compare continuous variables between two groups. A bivariate analysis of the categorical variables was carried out using a bivariate logistic regression analysis. The clinically important variables were included in this model regardless of their *P*‐values. A *P*‐value of <0.05 was considered to indicate statistical significance. All of the statistical analyses were undertaken using R (version 3.2.2; R Foundation for Statistical Computing, Vienna, Austria).

## Results

### Patient characteristics

**Figure 1:**
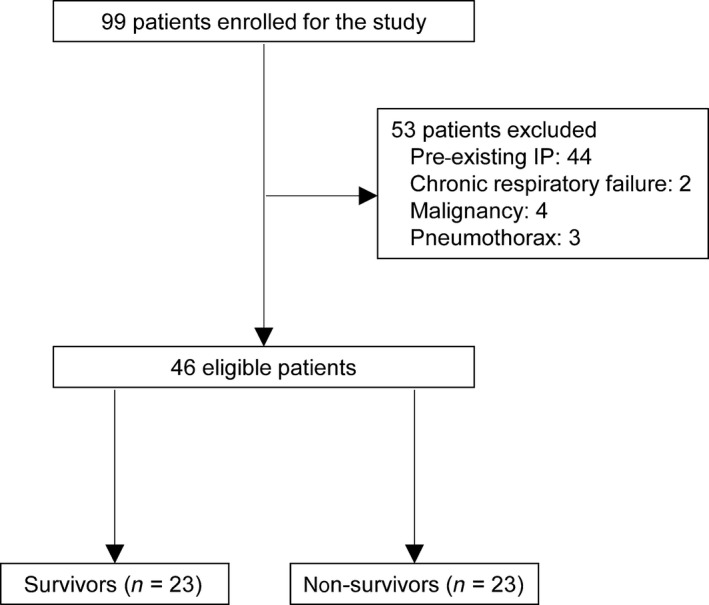
Flow diagram showing the steps of case selection. IP, interstitial pneumonia.

A total of 46 patients were eligible for this study, and were classified into two groups, survivors (*n* = 23) and non‐survivors (*n* = 23) (Fig. 1). The patient characteristics are summarized in Table 1. In comparison to non‐survivors, survivors showed lower neutrophil counts and serum Kerbs von Lungren‐6 antigen levels, and higher serum C‐reactive protein levels; however, these differences did not reach statistical significance. The other variables of the survivors and non‐survivors were comparable. The cause of death in the non‐survivors (*n* = 23) were: respiratory failure (*n* = 16), multiple organ failure (*n* = 5), disseminated intravascular coagulation (*n* = 1), and systemic cytomegalovirus infection (*n* = 1).

**Table 1:** Characteristics of patients with acute respiratory distress syndrome lacking common risk factors who were treated with high‐dose steroid therapy, grouped according to survival at 60 days after initiation of therapy

Variables	Survivors	Non‐survivors	*P*‐value
Patients, *n*	23	23	
Age, years	74 (70–81)	74 (71–82)	0.95
Gender, male / female	17/6	16/7	1.00
Smoking history	18 (78.2)	14 (60.9)	0.34
Respiratory rate, /min	21 (18.5–24.0)	20 (13–24)	0.46
PaCO_2_, mmHg	34.8 (31.7–39.4)	36.2 (32.8–40.5)	0.47
P/F ratio	177 (120–200)	166 (126–200)	0.84
APACHE II score	11 (9–12)	11.5 (9.75–13.5)	0.24
SOFA score	3 (3–4)	3 (3–4)	0.74
SOFA score except for respiratory parameter	0 (0–1)	0 (0–1)	0.82
Comorbidities			
COPD	2 (8.70)	2 (8.70)	1.00
Cancer	10 (43.5)	11 (47.8)	1.00
Diabetes mellitus	3 (13.0)	5 (21.7)	0.70
Laboratory data			
WBC, /μL	10,500 (7,950–14,250)	11,900 (9,100–13,100)	0.85
Neutrophil, /μL	8,050 (5,620–12,700)	9,990 (8,230–11,700)	0.69
KL‐6, U/mL (*n* = 43)	754 (410–1,320)	905 (515–1,590)	0.39
SP‐A, ng/mL (*n* = 20)	120 (91.5–144)	107 (94.8–198)	0.91
SP‐D, ng/mL (*n* = 29)	439 (184–569)	497 (295–689)	0.59
LDH, U/L	382 (328–502)	377 (292–489)	0.37
CRP, mg/dL	12.0 (6.16–16.1)	9.7 (6.70–19.5)	0.97

Data are expressed as the group median (interquartile range) or number (%).

APACHE II, Acute Physiology and Chronic Health Evaluation II; COPD, chronic obstructive pulmonary disease; CRP, C‐reactive protein; KL‐6, Kerbs von Lungren‐6 antigen; LDH, lactate dehydrogenase; P/F, PaO_2_/F_I_O_2_; SOFA, Sequential Organ Failure Assessment; SP, surfactant protein; WBC, white blood cell count.

### Concurrent treatments

The concurrent treatments during 60 days after the initiation of MPPT are summarized in Table 2. The frequency of concurrent therapies did not differ between the survivors and non‐survivors with statistical significance.

**Table 2:** Treatments given concurrently to patients with acute respiratory distress syndrome lacking common risk factors during 60 days after the initiation of high‐dose steroid therapy

Variables	Survivors	Non‐survivors	*P*‐value
Patients, *n*	23	23	
Mechanical ventilation	8 (34.8)	12 (51.7)	0.37
Antimicrobial agents	23 (100)	22 (95.7)	1.00
Immunosuppressants	5 (21.7)	4 (17.4)	1.00
Sivelestat sodium hydrate	2 (8.70)	5 (21.7)	0.41
rh TM	1 (4.35)	3 (13.0)	0.61

Data are expressed as number (%).

rh TM, recombinant human soluble thrombomodulin.

### Etiology and histocytological appearance

The etiologies of ARDS lacking common risk factors are listed in Table 3. As shown in Table 3, there were more survivors than non‐survivors in each of the subgroups of CTD‐ILD and drug‐induced lung injury; however, the differences were not statistically significant. Furthermore, although the non‐survivors outnumbered the survivors in the subgroup of “unknown”, there was no significant difference.

**Table 3:** Etiologies of acute respiratory distress syndrome lacking common risk factors, grouped according to patient survival at 60 days after initiation of high‐dose steroid therapy

Variables	Total	Survivors	Non‐survivors	*P*‐value
Patients, *n*	46	23	23	
CTD‐ILD	7 (15.2)	4 (17.4)	3 (13.0)	1.00
Rheumatoid arthritis	3	3	0	
Polymyositis/dermatomyositis	2	0	2	
Sjögren's syndrome	1	0	1	
Microscopic polyarteritis	1	1	0	
Drug‐induced lung injury	17 (37.0)	10 (43.5)	7 (30.4)	0.54
Cytotoxic anticancer agents	10	6	4	
Molecular‐targeted drugs	4	1	3	
Antibacterial agents	2	2	0	
Chinese herbal medicine	1	1	0	
Unknown	22 (47.8)	9 (39.1)	13 (56.5)	0.38

Data are expressed as number (%).

CTD‐ILD, connective tissue disease‐associated interstitial lung disease.

A histocytological diagnosis was established after the initiation of MPPT in 12 patients (26.1%). Four of these patients were diagnosed with DAD at autopsy, three patients were diagnosed with organizing pneumonia, and one patient was diagnosed with hypersensitivity pneumonia by transbronchial lung biopsy. Three patients were diagnosed with diffuse alveolar hemorrhage and one patient was diagnosed with eosinophilic pneumonia by a combination of bronchoalveolar lavage fluid analysis with radiological or laboratory findings.

### Chest CT

**Figure 2:**
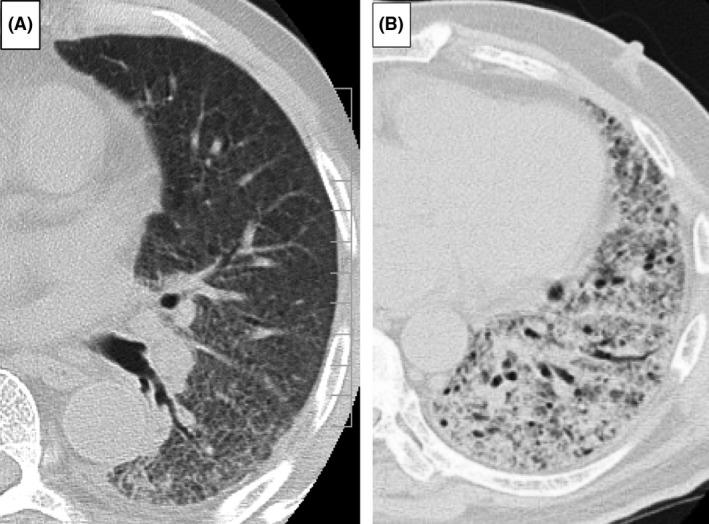
Representative chest high‐resolution computed tomography (CT) images of patients with acute respiratory distress syndrome lacking common risk factors who were treated with high‐dose steroid therapy. A, Chest high‐resolution CT in an 81‐year‐old man alive 60 days after initiation of therapy (survivor) showing ground‐glass attenuation predominance. B, Chest high‐resolution CT in a 76‐year‐old male non‐survivor showing consolidation predominance with bronchial dilatation.

A summary of the CT findings and the time of CT examination is shown in Table 4. The time of CT examination was comparable between the groups. The extent of GGA in the lungs of survivors was higher than that in non‐survivors; however, the difference did not reach statistical significance. The extent of consolidation in survivors (2.50% [0.83–4.75]) was significantly lower than that of non‐survivors (9.17% [2.71–14.0]) but the difference between the two groups was small (6.67%). Notably, the percentage of consolidation/(GGA + consolidation) in survivors was significantly lower than that in non‐survivors (survivors, 5.63% [2.31–13.8] versus non‐survivors, 27.2% [5.97–41.4]; *P *=* *0.01). Representative CT findings in survivors and non‐survivors are shown in Figure 2.

**Table 4:** Computed tomography (CT) findings and the time of CT examination in patients with acute respiratory distress syndrome (ARDS) lacking common risk factors, grouped according to survival at 60 days after initiation of high‐dose methylprednisolone pulse therapy (MPPT)

Variables	Total	Survivors	Non‐survivors	*P*‐value
Patients, *n*	46	23	23	
Time of CT examination, days				
From the onset of ARDS	0 (−1 to 0)	0 (−0.5 to 0)	0 (−1.5 to 0.5)	0.91
From the start of MPPT	0 (−2 to 0)	0 (−1 to 0)	0 (−2 to 0)	0.54
CT findings				
GGA, %	35.4 (18.6–51.0)	37.9 (29.0–57.1)	25.8 (17.1–47.5)	0.17
CO, %	4.07 (1.46–11.7)	2.50 (0.83–4.75)	9.17 (2.71–14.0)	0.02
GGA + CO, %	43.8 (33.3–58.2)	46.7 (32.3–58.8)	37.5 (33.3–56.7)	0.69
CO/(GGA + CO), %	10.0 (3.46–28.4)	5.63 (2.31–13.8)	27.2 (5.97–41.4)	0.01
Bronchial dilation	42 (91.3)	20 (87.0)	22 (95.7)	0.61
Honeycombing	4 (8.70)	3 (13.0)	1 (4.35)	0.61
Interlobular septal thickening	23 (50.0)	13 (56.5)	10 (43.5)	0.56
Pleural effusion	21 (45.7)	10 (43.5)	11 (47.8)	1.00

Data are expressed as the group median (interquartile range) or number (%).

CO, consolidation; GGA, ground‐glass attenuation.

### Predictors of 60‐day survival

A summary of the results of the bivariate analysis of prognostic factors for 60‐day survival is shown in Table 5. The percentage of consolidation/(GGA + consolidation) on chest CT was significantly associated with a 60‐day survival (odds ratio 0.97; 95% confidence interval, 0.93–0.99; *P *=* *0.042). When stratified by variables of age, P/F ratio, SOFA score, and APACHE II score, odds ratios of the percentage of consolidation/(GGA + consolidation) were significant in the adjusted models.

**Table 5:** Association of the percentage of consolidation/(ground‐glass attenuation + consolidation) with 60‐day survival after initiation of high‐dose methylprednisolone pulse therapy in patients with acute respiratory distress syndrome lacking common risk factors

Model	OR (95% CI)	*P*‐value
Unadjusted	0.97 (0.93–0.99)	0.042
Adjusted for		
Age	0.97 (0.94–0.99)	0.042
P/F ratio	0.97 (0.94–1.00)	0.041
SOFA score	0.97 (0.93–0.99)	0.044
APACHE II score	0.95 (0.91–0.99)	0.011

APACHE II, Acute Physiology and Chronic Health Evaluation II; CI, confidence interval; P/F, PaO_2_/F_I_O_2_; OR, odds ratio; SOFA, Sequential Organ Failure Assessment.

## Discussion

Pure ARDS/DAD is usually resistant to treatment, but ARDS lacking common risk factors sometimes shows responsiveness to corticosteroid treatment.^4^, ^5^, ^6^, ^8^, ^19^, ^20^ Although the histological diagnosis of acute pulmonary injury is therefore quite important,^8^ invasive procedures cannot be applied in all patients with acute respiratory failure. Actually, we could only perform antemortem pathological examinations in 8/46 (17.4%) patients. We found that the low percentage of consolidation/(GGA + consolidation) on the chest CT scans was associated with a significant increase in 60‐day survival in patients with ARDS lacking common risk factors after high‐dose steroid therapy.

High‐resolution CT is commonly used as an alternative non‐invasive procedure to assist in reaching a histological diagnosis. However, previous studies showed that consolidation and GGA are not helpful for predicting the specific underlying histology in diffuse infiltrative lung diseases.^21^, ^22^ We speculated that the percentage of consolidation/(GGA + consolidation) might be helpful to separate histological DAD pattern of pulmonary injury from non‐DAD pattern of pulmonary injury. Patients with DAD show a mixture of consolidation and diffuse GGA on high‐resolution CT.^23^ Johkoh *et al*.^24^ reported that idiopathic DAD showed airspace consolidation involving 25 ± 15% (mean ± standard deviation) of the lung parenchyma and GGA involving 53 ± 18% of the lung parenchyma. The calculated percentage of consolidation/(GGA + consolidation) in the study of Johkoh *et al*. was obviously higher (mean 28.4%) than that in our study cohort (median, 10.0%). Although the methodology to evaluate the CT findings in this study was similar to that in the study by Johkoh *et al*., the study population was different: mixture of DAD and non‐DAD (our study) versus only DAD (Johkoh *et al*.). Therefore, the difference of the percentage of consolidation/(GGA + consolidation) between the two studies might be due to the difference in study cohorts.

It can be also speculated that the difference of radiological appearances might simply reflect disease progression or improvement of lung injury because the findings of chest CT could change according to the phase of lung injury in patients with ARDS.^25^ However, this speculation is also unlikely in this study because the period from the onset of ARDS to the CT examination was comparable between the groups in the present study. We are therefore of the opinion that survival might be influenced not only by disease severity but also by a corticosteroid sensitivity in this study.

The present study is associated with several limitations. First, it was a single‐center retrospective study and was subjected to selection bias. In addition, the small sample size caused some analyses to be under‐powered. Second, 11/23 (47.8%) non‐survivors were not treated with mechanical ventilation. The treatment of these patients might therefore have been inadequate in some cases. Third, it is unknown whether MPPT truly led to some patients’ survival because we could not compare the survival between MPPT‐treated patients and control patients who did not receive MPPT. Finally, we included patients who did not receive positive‐pressure mechanical ventilation but who had a P/F ratio of ≤300. The definition of ARDS recommends examining the P/F ratio under positive end‐expiratory pressure or continuous positive airway pressure, and the measurement of a P/F ratio without such mechanical ventilation can be unreliable.

## Conclusions

To the best of the authors’ knowledge, this is the first study to evaluate the prognostic factors in patients with ARDS lacking common risk factors after high‐dose steroid therapy. The percentage of the extent of consolidation/(GGA + consolidation) was significantly associated with 60‐day survival of this population.

## Disclosure

Approval of the research protocol: The protocol for this research project has been approved by a suitably constituted Ethics Committee of the institution and it conforms to the provisions of the Declaration of Helsinki. Committee of the Fukuoka University Hospital Institutional Review Board, Approval No. 16‐1‐15. Ethics Committee of the institution waived the requirement for informed consent.

Conflict of interest: None.

## References

[1] Definition Task Force ARDS , Ranieri VM , Rubenfeld GD *et al* Acute respiratory distress syndrome: the Berlin Definition. JAMA 2012; 307: 2526–33. 22797452 10.1001/jama.2012.5669

[2] Bernard GR , Artigas A , Brigham KL *et al* The American‐European Consensus Conference on ARDS. Definitions, mechanisms, relevant outcomes, and clinical trial coordination. Am. J. Respir. Crit. Care Med. 1994; 149: 818–24. 7509706 10.1164/ajrccm.149.3.7509706

[3] Gibelin A , Parrot A , Maitre B *et al* Acute respiratory distress syndrome mimickers lacking common risk factors of the Berlin definition. Intensive Care Med. 2016; 42: 164–72. 26408150 10.1007/s00134-015-4064-y

[4] Schwarz MI , Albert RK . “Imitators” of the ARDS: implications for diagnosis and treatment. Chest 2004; 125: 1530–5. 15078770 10.1378/chest.125.4.1530

[5] Guérin C , Thompson T , Brower R . The ten diseases that look like ARDS. Intensive Care Med. 2015; 41: 1099–102. 25527375 10.1007/s00134-014-3608-x

[6] de Prost N , Pham T , Carteaux G *et al* Etiologies, diagnostic work‐up and outcomes of acute respiratory distress syndrome with no common risk factor: a prospective multicenter study. Ann. Intensive Care 2017; 7: 69. 28631088 10.1186/s13613-017-0281-6 PMC5476531

[7] Peter JV , John P , Graham PL *et al* Corticosteroids in the prevention and treatment of acute respiratory distress syndrome (ARDS) in adults: meta‐analysis. BMJ 2008; 336: 1006–9. 18434379 10.1136/bmj.39537.939039.BE PMC2364864

[8] Patel SR , Karmpaliotis D , Ayas NT *et al* The role of open‐lung biopsy in ARDS. Chest 2004; 125: 197–202. 14718441 10.1378/chest.125.1.197

[9] Zhang Z , Chen L , Ni H . The effectiveness of Corticosteroids on mortality in patients with acute respiratory distress syndrome or acute lung injury: a secondary analysis. Sci. Rep. 2015; 5: 17654. 26627982 10.1038/srep17654 PMC4667272

[10] Riviello ED , Kiviri W , Twagirumugabe T *et al* Hospital incidence and outcomes of the acute respiratory distress syndrome using the Kigali modification of the Berlin definition. Am. J. Respir. Crit. Care Med. 2016; 193: 52–9. 26352116 10.1164/rccm.201503-0584OC

[11] Shiboski SC , Shiboski CH , Criswell LA *et al* American College of Rheumatology classification criteria for Sjögren's syndrome: a data‐driven, expert consensus approach in the Sjögren's International Collaborative Clinical Alliance cohort. Arthritis Care Res. (Hoboken) 2012; 64: 475–87. 22563590 10.1002/acr.21591 PMC3349440

[12] Bohan A , Peter JB . Polymyositis and dermatomyositis (second of two parts). N. Engl. J. Med. 1975; 292: 403–7. 1089199 10.1056/NEJM197502202920807

[13] Bohan A , Peter JB . Polymyositis and dermatomyositis (first of two parts). N. Engl. J. Med. 1975; 292: 344–7. 1090839 10.1056/NEJM197502132920706

[14] Arnett FC , Edworthy SM , Bloch DA *et al* The American Rheumatism Association 1987 revised criteria for the classification of rheumatoid arthritis. Arthritis Rheum. 1988; 31: 315–24. 3358796 10.1002/art.1780310302

[15] Jennette JC , Falk RJ , Andrassy K *et al* Nomenclature of systemic vasculitides. Proposal of an international consensus conference. Arthritis Rheum. 1994; 37: 187–92. 8129773 10.1002/art.1780370206

[16] Dhokarh R , Li G , Schmickl CN *et al* Drug‐associated acute lung injury: a population‐based cohort study. Chest 2012; 142: 845–50. 22539646 10.1378/chest.11-2103 PMC3465105

[17] Hansell DM , Bankier AA , MacMahon H *et al* Fleischner Society: glossary of terms for thoracic imaging. Radiology 2008; 246: 697–722. 18195376 10.1148/radiol.2462070712

[18] Ichikado K , Muranaka H , Gushima Y *et al* Fibroproliferative changes on high‐resolution CT in the acute respiratory distress syndrome predict mortality and ventilator dependency: a prospective observational cohort study. BMJ Open 2012; 2: e000545. 10.1136/bmjopen-2011-000545 PMC3293132 22382117

[19] Katzenstein AL , Bloor CM , Leibow AA . Diffuse alveolar damage–the role of oxygen, shock, and related factors. A review. Am. J. Pathol. 1976; 85: 209–28. 788524 PMC2032554

[20] Kaarteenaho R , Kinnula VL . Diffuse alveolar damage: a common phenomenon in progressive interstitial lung disorders. Pulm. Med. 2011; 2011: 1–10. 10.1155/2011/531302 PMC3099744 21637367

[21] Remy‐Jardin M , Giraud F , Remy J *et al* Importance of ground‐glass attenuation in chronic diffuse infiltrative lung disease: pathologic‐CT correlation. Radiology 1993; 189: 693–8. 8234692 10.1148/radiology.189.3.8234692

[22] Leung AN , Miller RR , Müller NL . Parenchymal opacification in chronic infiltrative lung diseases: CT‐pathologic correlation. Radiology 1993; 188: 209–14. 8511299 10.1148/radiology.188.1.8511299

[23] Sverzellati N , Lynch DA , Hansell DM *et al* American Thoracic Society‐European Respiratory Society classification of the idiopathic interstitial pneumonias: advances in knowledge since 2002. Radiographics 2013; 35: 1849–71. 10.1148/rg.2015140334 26452110

[24] Johkoh T , Müller NL , Taniguchi H *et al* Acute interstitial pneumonia: thin‐section CT findings in 36 patients. Radiology 1999; 211: 859–63. 10352616 10.1148/radiology.211.3.r99jn04859

[25] Zompatori M , Ciccarese F , Fasano L . Overview of current lung imaging in acute respiratory distress syndrome. Eur. Respir. Rev. 2014; 23: 519–30. 25445951 10.1183/09059180.00001314 PMC9487404

